# Supplementary material for: Robust SARS-CoV-2 Antibody Responses in Asian COVID-Naïve Subjects 180 Days after Two Doses of BNT162b2 mRNA COVID-19 Vaccine
Source: Vaccines (Basel). 2021 Oct 25;9(11):1241. doi: 10.3390/vaccines9111241 (PMC8622758; doi:10.3390/vaccines9111241)
Supplement: Supplementary file 1 [file vaccines-09-01241-s001.zip › vaccines-1418978-supplementary.pdf]

Supplementary Table S1: STARD checklist

Section & Topic	No	Item	Reported on page #
**TITLE OR ABSTRACT**			
	**1**	Identification as a study of diagnostic accuracy using at least one measure of accuracy (such as sensitivity, specificity, predictive values, or AUC)	**2**
**ABSTRACT**			
	**2**	Structured summary of study design, methods, results, and conclusions (for specific guidance, see STARD for Abstracts)	**2**
**INTRODUCTION**			
	**3**	Scientific and clinical background, including the intended use and clinical role of the index test	**4**
	**4**	Study objectives and hypotheses	**5**
**METHODS**			
Study design	**5**	Whether data collection was planned before the index test and reference standard were performed (prospective study) or after (retrospective study)	**6**
Participants	**6**	Eligibility criteria	**6**
	**7**	On what basis potentially eligible participants were identified (such as symptoms, results from previous tests, inclusion in registry)	**6**
	**8**	Where and when potentially eligible participants were identified (setting, location and dates)	**6**
	**9**	Whether participants formed a consecutive, random or convenience series	**6**
Test methods	**10a**	Index test, in sufficient detail to allow replication	**6**
	**10b**	Reference standard, in sufficient detail to allow replication	**7**
	**11**	Rationale for choosing the reference standard (if alternatives exist)	**7**
	**12a**	Definition of and rationale for test positivity cut-offs or result categories of the index test, distinguishing pre-specified from exploratory	**7**
	**12b**	Definition of and rationale for test positivity cut-offs or result categories of the reference standard, distinguishing pre-specified from exploratory	**7**
	**13a**	Whether clinical information and reference standard results were available to the performers/readers of the index test	**7**
	**13b**	Whether clinical information and index test results were available to the assessors of the reference standard	**7**
Analysis	**14**	Methods for estimating or comparing measures of diagnostic accuracy	**8**
	**15**	How indeterminate index test or reference standard results were handled	**8**
	**16**	How missing data on the index test and reference standard were handled	**8**
	**17**	Any analyses of variability in diagnostic accuracy, distinguishing pre-specified from exploratory	**8**
	**18**	Intended sample size and how it was determined	**Not applicable**
**RESULTS**			
Participants	**19**	Flow of participants, using a diagram	**Not applicable**
	**20**	Baseline demographic and clinical characteristics of participants	**6**
	**21a**	Distribution of severity of disease in those with the target condition	**Not applicable**
	**21b**	Distribution of alternative diagnoses in those without the target condition	**Not applicable**
	**22**	Time interval and any clinical interventions between index test and reference standard	**Not applicable**
Test results	**23**	Cross tabulation of the index test results (or their distribution) by the results of the reference standard	**9**
	**24**	Estimates of diagnostic accuracy and their precision (such as 95% confidence intervals)	**Not applicable**

	**25**	Any adverse events from performing the index test or the reference standard	**Not applicable**
**DISCUSSION**			
	**26**	Study limitations, including sources of potential bias, statistical uncertainty, and generalisability	**17**
	**27**	Implications for practice, including the intended use and clinical role of the index test	**16**
**OTHER INFORMATION**			
	**28**	Registration number and name of registry	**8**
	**29**	Where the full study protocol can be accessed	**Not applicable**
	**30**	Sources of funding and other support; role of funders	**Not applicable**

Supplementary Table S2: IgM values at various time points after first and second vaccination.

Time point	n	Median (COI)	Range
Pre-vaccination	70	0.030000	0.01 – 0.77
10 days post-dose 1	71	0.36000	0.02 – 5.33
20-40 days post-dose 1	8	0.55857	0.11 – 7.55
20 days post-dose 2	67	2.26000	0.13 – 13.6
40 days post-dose 2	48	1.34996	0.09 – 15.8
60 days post-dose 2	49	0.86000	0.07 – 2.96
90 days post-dose 2	37	0.41000	0.07 – 1.99
120 days post-dose 2	8	0.10954	0.04 – 1.92
150 days post-dose 2	18	0.15969	0.02 – 1.05
180 days post-dose 2	8	0.13416	0.03 – 0.83

Supplementary Table S3: Antibody comparison of titres >90 days post-second vaccination between subjects <50 and ≥50 years old

	**120 days**	**150 days**	**180 days**	**All**
**N <50/≥50**	4/4	9/9	5/3	18/16
**T-Ab (BAU/mL)**				
<50 Median, Range	1999 (1587-2852)	1327 (415-2852)	664 (180-2936)	1415 (180-2936)
≥50 Median, Range	1129 (370-2125)	765 (357-1803)	1294 (344-1594)	814 (344-2125)
Median difference, p	1091, p = 0.20	463, p = 0.11	164, p = 0.79	414, p = 0.10
**IgG (BAU/mL)**				
<50 Median, Range	454 (440-669)	307 (75-445)	217 (88-2452)	355 (75-2452)
≥50 Median, Range	377 (105-733)	179 (75-450)	310 (80-314)	210 (75-733)
Median difference, p	177, p = 0.89	118, p = 0.14	115, p = 0.79	123, p = 0.09
**IgM (COI)**				
<50 Median, Range	0.83 (0.08-1.92)	0.28 (0.06-0.66)	0.18 (0.04-0.31)	0.22 (0.04-1.92)
≥50 Median, Range	0.11 (0.04-1.25)	0.12 (0.02-1.05)	0.10 (0.03-0.83)	0.12 (0.02-1.25)
Median difference, p	0.19, p = 0.25	0.06, p = 0.31	0.01, p = 1.00	0.05, p = 0.22
**N-Ab (ug/mL)**				
<50 Median, Range	2.53 (1.93-2.82)	1.70 (0.43-2.82)	1.13 (0.66-1.53)	1.67 (0.43-2.82)
≥50 Median, Range	0.61 (0.58-2.46)	1.06 (0.39-2.36)	1.73 (0.39-1.92)	1.06 (0.39-2.46)
Median difference, p	1.57, p = 0.23	0.64, p = 0.09	0.55, p = 0.57	0.47, p = 0.06

Supplementary Table S4: Comparison of all antibody titres >90 days post-second vaccination between male and female subjects

**N Male/Female**	9/25
**T-Ab (BAU/mL)**	
Male Median, Range	762, 344-2851
Female Median, Range	1269, 180-2936
Median difference, p	84, p = 0.80
**IgG (BAU/mL)**	
Male Median, Range	246, 80-445
Female Median, Range	307, 75-2452
Median difference, p	61, p = 0.49
**IgM (COI)**	
Male Median, Range	0.08, 0.02-1.25
Female Median, Range	0.17, 0.03-1.92
Median difference, p	0.03, p = 0.62
**N-Ab (ug/mL)**	
Male Median, Range	1.31, 0.39-2.82
Female Median, Range	1.51, 0.39-2.67
Median difference, p	0.06, p = 0.82
